# The Aging Brain: Crossroad of Normal Aging and Dementia

**DOI:** 10.1093/geroni/igaa057.2767

**Published:** 2020-12-16

**Authors:** Qu Tian, Luigi Ferrucci

## Abstract

The functional consequences of the aging brain include several aspects of physical and cognitive decline that ultimately cause loss of mobility and dementia. Although in certain individuals, the cognitive and physical correlates of the aging brain occur in parallel, others show decline in one of these functional parameters. Underlying mechanism of this complex process that lead to different manifestations is not well understood. Proposed mechanisms include brain structural changes, tau pathology, specific white matter degeneration, metabolic derangement mostly including lipids metabolism and others. This symposium aims to address the complexity of the aging brain by showcasing studies that span the continuum from normal aging to dementia using data from the Baltimore Longitudinal Study of Aging (BLSA). The wealth of neuroimaging and phenotype data from the BLSA provides the unique opportunity to investigate neural substrates and predictors of aging phenotype, and mechanisms of age-related neurodegeneration and pathology, such as dementia, and loss of mobility. First, we identify multimodal neuroimaging predictors of important aging phenotypes of gait decline (Sargent/Tian) and memory decline (Bilgel). Second, using advanced quantitative MRI technology, we investigate underlying mechanisms of age-related white matter degeneration through potential oligodendrocyte metabolism (Bouhrara). Third, we demonstrate unique cognitive and neuroimaging profiles of dual memory and gait decline (Tian) and neural substrates for bile acids, the primary cholesterol breakdown products (Varma) in relation to dementia. We seek to generate discussions of mechanisms of the aging brain that connect the age-related phenotypes, such as decline of mobility and cognition, to the development of dementia.

